# Perioperative Systemic Therapy Versus Cytoreductive Surgery and HIPEC Alone for Resectable Colorectal Peritoneal Metastases: Patient-Reported Outcomes of a Randomized Phase II Trial

**DOI:** 10.1245/s10434-023-13116-z

**Published:** 2023-02-08

**Authors:** C. Bakkers, K. P. Rovers, A. Rijken, G. A. A. M. Simkens, C. S. Bonhof, S. W. Nienhuijs, J. W. A. Burger, G. J. M. Creemers, A. R. M. Brandt-Kerkhof, J. B. Tuynman, A. G. J. Aalbers, M. J. Wiezer, P. R. de Reuver, W. M. U. van Grevenstein, P. H. J. Hemmer, C. J. A. Punt, P. J. Tanis, F. Mols, I. H. J. T. de Hingh, the Dutch Peritoneal Oncology Group and the Dutch Colorectal Cancer Group, A. M. J. Thijs, R. J. Lurvink, E. V. E. Madsen, E. van Meerten, M. Kusters, K. S. Versteeg, N. F. M. Kok, T. E. Buffart, D. Boerma, M. Los, J. H. W. de Wilt, H. M. W. Verheul, S. Kruijff, D. J. A. de Groot, M. Koopman

**Affiliations:** 1 Department of Surgery, Catharina Cancer Institute, Eindhoven, The Netherlands; 2 Center of Research on Psychological and Somatic Disorders, Department of Medical and Clinical Psychology, Tilburg University, Tilburg, The Netherlands; 3 Department of Research, Netherlands Comprehensive Cancer Organization (IKNL), Utrecht, The Netherlands; 4 Department of Medical Oncology, Catharina Cancer Institute, Eindhoven, The Netherlands; 5 Department of Surgery, Erasmus University Medical Center, Rotterdam, The Netherlands; 6 Department of Surgery, Amsterdam University Medical Centers, VUMC, Amsterdam, The Netherlands; 7 Department of Surgery, Netherlands Cancer Institute, Amsterdam, The Netherlands; 8 Department of Surgery, St. Antonius Hospital, Nieuwegein, The Netherlands; 9 Department of Surgery, Radboud University Medical Center, Nijmegen, The Netherlands; 10 Department of Surgery, University Medical Center Utrecht, Utrecht, The Netherlands; 11 Department of Surgery, University Medical Center Groningen, Groningen, The Netherlands; 12 Julius Center for Health Sciences and Primary Care, University Medical Center Utrecht, Utrecht, The Netherlands; 13 Department of Surgery, Amsterdam University Medical Centers, University of Amsterdam, Cancer Center Amsterdam, Amsterdam, The Netherlands; 14 GROW – School for Oncology and Development Biology, Maastricht University, Maastricht, The Netherlands

## Abstract

**Background:**

As part of a randomized phase II trial in patients with isolated resectable colorectal peritoneal metastases (CPMs), the present study compared patient-reported outcomes (PROs) of patients treated with perioperative systemic therapy versus cytoreductive surgery and hyperthermic intraperitoneal chemotherapy (CRS–HIPEC) alone. Also, PROs of patients receiving perioperative systemic therapy were explored.

**Patients and Methods:**

Eligible patients were randomized to perioperative systemic therapy (experimental) or CRS–HIPEC alone (control). PROs were assessed using EORTC QLQ-C30, QLQ-CR29, and EQ-5D-5L questionnaires at baseline, after neoadjuvant treatment (experimental), and at 3 and 6 months postoperatively. Linear mixed modeling was used to compare five predefined PROs (visual analog scale, global health status, physical functioning, fatigue, C30 summary score) between arms and to longitudinally analyze PROs in the experimental arm.

**Results:**

Of 79 analyzed patients, 37 (47%) received perioperative systemic therapy. All predefined PROs were comparable between arms at all timepoints and returned to baseline at 3 or 6 months postoperatively. The experimental arm had worsening of fatigue [mean difference (MD) + 14, *p* = 0.001], loss of appetite (MD + 15, *p* = 0.003), hair loss (MD + 18, *p* < 0.001), and loss of taste (MD + 27, *p* < 0.001) after neoadjuvant treatment. Except for loss of appetite, these PROs returned to baseline at 3 or 6 months postoperatively.

**Conclusions:**

In patients with resectable CPM randomized to perioperative systemic therapy or CRS–HIPEC alone, PROs were comparable between arms and returned to baseline postoperatively. Together with the trial’s previously reported feasibility and safety data, these findings show acceptable tolerability of perioperative systemic therapy in this setting.

**Supplementary Information:**

The online version contains supplementary material available at 10.1245/s10434-023-13116-z.

Cytoreductive surgery (CRS) with or without hyperthermic intraperitoneal chemotherapy (HIPEC) has been recommended for selected patients with resectable colorectal peritoneal metastases in the majority of (inter)national guidelines.^1^ Little is known about the value of perioperative systemic therapy for resectable colorectal peritoneal metastases in the absence of randomized trials,^2^ leading to a wide variety in its administration among countries and hospitals.^1–3^ To address this evidence gap, the CAIRO6 trial randomizes these patients to perioperative (i.e., neoadjuvant and adjuvant) systemic therapy and CRS–HIPEC or CRS–HIPEC alone.^4^ Although superior survival of perioperative systemic therapy is hypothesized,^4^ it prolongs and intensifies treatment, may lead to (sometimes severe) toxicity,^5^ could increase postoperative morbidity (especially when including bevacizumab^6^), and may result in preoperative intraperitoneal progression and consequent inoperability given its assumed relative inefficacy for colorectal peritoneal metastases.^7^ Altogether, this could also worsen patient-reported outcomes (PROs). To address these issues, CAIRO6 incorporated a randomized phase II trial to assess the feasibility, safety, and PROs of perioperative systemic therapy in this setting.^4,8^ As part of this phase II trial, the present study aimed to compare PROs between both treatment arms. A secondary aim was to longitudinally explore PROs of patients receiving perioperative systemic therapy.

## Patients and Methods

### Design

CAIRO6 is an investigator-initiated, parallel-group, open-label, phase II–III, randomized, superiority trial conducted in all nine Dutch tertiary hospitals for the surgical treatment of colorectal peritoneal metastases. The trial is approved by a central ethics committee (MEC-U, Nieuwegein, the Netherlands, R16.056) and the institutional review boards of all participating hospitals. The trial is registered (Clinicaltrials.gov: NCT02758951). The trial protocol^4^ and the feasibility and safety data of the phase II trial (i.e., mortality, morbidity, surgical details, hospital stay)^8^ have been previously published. Therefore, only brief descriptions of eligible patients, randomization procedures, and interventions are provided.

### Patients

Eligible patients were adults with a World Health Organization performance status of 0–1, pathologically proven isolated resectable colorectal peritoneal metastases, no systemic therapy for colorectal cancer within 6 months prior to enrolment, and no previous CRS–HIPEC.^4,8^ All patients gave written informed consent.

### Randomization

Patients were randomized 1:1 to perioperative systemic therapy (experimental arm) or CRS–HIPEC alone (control arm) using minimization stratified by previous systemic therapy for colorectal cancer (yes, no), onset of peritoneal metastases (synchronous, metachronous), peritoneal cancer index (≤ 10, > 10), and planned HIPEC regimen (mitomycin C, oxaliplatin).

### Interventions

#### Perioperative Systemic Therapy

At physician’s discretion, perioperative systemic therapy comprised either six two-weekly neoadjuvant and six two-weekly adjuvant cycles of FOLFOX (5-fluorouracil, leucovorin, oxaliplatin), four three-weekly neoadjuvant and four three-weekly adjuvant cycles of CAPOX (capecitabine, oxaliplatin), or six two-weekly neoadjuvant cycles of FOLFIRI (5-fluorouracil, leucovorin, irinotecan) followed by either six two-weekly adjuvant cycles of 5-fluorouracil with leucovorin or four three-weekly adjuvant cycles of capecitabine.^4,8^ Bevacizumab was added to the first three (CAPOX) or four (FOLFOX or FOLFIRI) neoadjuvant cycles.^4,8^ In case of unacceptable toxicity, it was allowed to switch from CAPOX or FOLFOX to FOLFIRI (and vice versa) during neoadjuvant treatment and to fluoropyrimidine monotherapy during adjuvant treatment. Perioperative systemic therapy was terminated in case of disease progression, unacceptable toxicity, patient’s request, or physician’s decision.

#### Surgery

CRS–HIPEC was performed according to the standardized Dutch protocol.^9^ CRS was performed only if macroscopic complete CRS was deemed achievable after explorative laparotomy. Only if macroscopic complete CRS was achieved, HIPEC was performed using mitomycin C or oxaliplatin according to local protocol.^9^ In case of unresectable disease or macroscopic incomplete CRS, trial treatment was stopped and patients were offered off-protocol palliative treatment.

### PRO Assessment

Patients were asked to give separate informed consent for PRO assessment. PROs were assessed using three validated questionnaires (EORTC QLQ-C30,^10^ EORTC QLQ-CR29,^11^ EuroQoL EQ-5D-5L^12^) before trial treatment, after completion of neoadjuvant treatment (experimental arm only), and 3 and 6 months after (intended) surgery. At patient’s preference, questionnaires were sent on paper or electronically using certified software (Research Manager, Deventer, the Netherlands). Supplementary Table S1 presents the PROs of each questionnaire. The manuals of EORTC and EuroQol were used to calculate scores for all PROs.^13–15^ In general, PROs can be divided into function scales (with higher scores indicating better functioning) or symptom scales (with higher scores indicating worse symptoms). For the primary study aim (i.e., comparison of PROs between both arms), five PROs were predefined by the investigators as the most appropriate to assess overall health and treatment tolerability: visual analog scale, global health status, physical functioning, fatigue, and C30 summary score. For the secondary study aim (i.e., longitudinal exploration of PROs of patients receiving perioperative systemic therapy in the experimental arm), all PROs were analyzed.

### Statistical Analysis

The investigators and the ethics committee agreed upon an a priori determined sample size of 80 patients for the phase II trial as a sufficient number to assess the feasibility and safety of perioperative systemic therapy.^4,8^ Given the explorative nature of PRO analyses, no PRO hypothesis was defined a priori. As the present study aimed to assess PROs of actual treatment rather than treatment assignment, analyses were done in a modified intention-to-treat PRO population of all patients starting neoadjuvant treatment (experimental arm) or undergoing upfront surgery (control arm). Statistical tests were performed two-sided using IBM SPSS Statistics (v25.0, IBM Corp, Armonk, NY, USA). Baseline characteristics of the modified intention-to-treat PRO population were compared between both arms using Student’s *t*-test or Mann–Whitney *U* test for continuous variables and chi-square test or Fisher’s exact test for categorical variables, with *p* < 0.05 being considered statistically significant for these comparisons.

For the primary study aim (i.e., comparison of five predefined PROs between both arms), all patients who completed questionnaires at two or more comparative timepoints (i.e., baseline and 3 and 6 months postoperatively) were included. In these patients, differential effects in scores over time and scores at each timepoint were compared between both arms using linear mixed modeling (LMM) with the use of maximum likelihood estimation and an unstructured covariance matrix with a two-level structure [i.e., repeated timepoints (lower level), patients (higher level)]. If there were no statistically significant differences in differential effects in scores over time and in scores at each timepoint, scores of both arms were merged to longitudinally compare baseline scores with scores at subsequent timepoints using LMM. To account for multiple testing in primary comparative analyses, *p* < 0.01 was considered statistically significant (Bonferroni correction: *p* < 0.05 divided by five main comparisons).

For the secondary study aim (i.e., longitudinal exploration of all PROs of patients receiving perioperative systemic therapy in the experimental arm), all patients who completed questionnaires at baseline and after neoadjuvant treatment were included. In these patients, baseline scores were compared with scores measured after neoadjuvant treatment using LMM. All PROs with a statistically significant difference in scores between these timepoints were further analyzed and (graphically) presented. To account for multiple testing in secondary explorative analyses, statistical significance was pragmatically set at *p* < 0.01.

For each statistically significant difference, a Cohen’s *d* (CD) effect size was calculated to assess its clinical relevance, with CD ≥ 0.5 being considered clinically relevant.^16^ Since means were used to determine effect sizes and to present differences, all PRO scores were presented as mean (standard deviation) regardless of distribution.

## Results

**Fig. 1:**
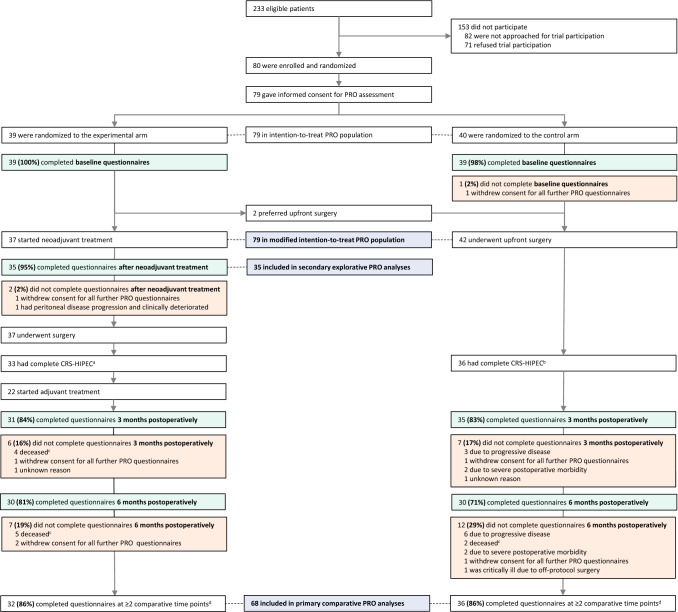
Patient pathway and response rates (including reasons for non-response) at all timepoints. *CRS–HIPEC* cytoreductive surgery and hyperthermic intraperitoneal chemotherapy, *PRO* patient-reported outcome. ^a^Reasons for discontinuation to CRS–HIPEC (experimental): four unresectable peritoneal metastases. ^b^Reasons for discontinuation to CRS–HIPEC (control): five unresectable peritoneal metastases, one unexpected liver metastases. ^c^All due to progressive disease (experimental + control). ^d^Baseline and 3 and 6 months postoperatively

Between 15 June 2017 and 9 January 2019, 233 patients were eligible for trial participation, 80 were randomized (40 to each arm, baseline characteristics of the intention-to-treat population in Supplementary Table S2), and 79 gave informed consent for PRO assessment (Fig. 1). The modified intention-to-treat PRO population comprised all these 79 patients, of whom 37 started neoadjuvant treatment (experimental arm) and 42 underwent upfront surgery (control arm) (Fig. 1). Table 1 presents the baseline characteristics of the modified intention-to-treat PRO population. The intention-to-treat population and the modified intention-to-treat PRO population had comparable distributions of baseline characteristics (Table 1, Supplementary Table S2).

**Table 1:** Baseline characteristics of the modified intention-to-treat PRO population

	Experimental arm (*n *= 37)	Control arm (*n *= 42)	*p* value
Sex, *n* (%)			0.333
Male	18 (49)	25 (60)	
Female	19 (51)	17 (40)	
Age in years, mean (SD)	59 (11)	64 (10)	**0.032**
WHO performance score, *n* (%)			0.331
0	27 (73)	35 (83)	
1	9 (24)	7 (17)	
2	1 (3)^a^	0 (0)	
Primary tumor location, *n* (%)			0.705
Proximal colon^b^	16 (43)	15 (36)	
Distal colon^c^	19 (51)	26 (62)	
Rectum	1 (3)	1 (2)	
Multiple	1 (3)	0 (0)	
Primary tumor status, *n* (%)			0.209
Resected	27 (73)	25 (60)	
In situ	10 (27)	17 (40)	
Previous adjuvant systemic chemotherapy for colorectal cancer, *n* (%)			0.743
No	27 (73)	32 (76)	
Yes	10 (27)	10 (24)	
Onset of peritoneal metastases, *n* (%)			0.783
Synchronous	20 (54)	24 (57)	
Metachronous	17 (46)	18 (43)	
Baseline peritoneal cancer index, median (range)	3 (0-15)	5 (0–18)	0.104
Planned HIPEC regimen, *n* (%)			0.988
Mitomycin C	30 (81)	34 (81)	
Oxaliplatin	7 (19)	8 (19)	

Statistically significant value given in bold

*HIPEC* hyperthermic intraperitoneal chemotherapy, *SD* standard deviation, *WHO* World Health Organization

^a^Due to severe obesity

^b^Cecum, ascending colon, hepatic flexure, transverse colon

^c^Splenic flexure, descending colon, sigmoid, rectosigmoid

Figure 1 presents the patient pathway and questionnaire response rates (including reasons for nonresponse) at each timepoint. Overall response rates were 99% (78 of 79 patients) at baseline, 95% (35 of 37 patients) after completion of neoadjuvant treatment (experimental arm), 84% (66 of 79 patients) at 3 months postoperatively, 76% (60 of 79 patients) at 6 months postoperatively, and 87% (239 of 274 timepoints) in the entire phase II trial. Response rates were comparable between both arms at all timepoints (data not shown). PRO scores of all patients at all timepoints are presented in Table 2. Primary comparative analyses and secondary explorative analyses were performed in 68 and 35 patients, respectively (Fig. 1). PRO scores of patients included in primary comparative analyses and secondary explorative analyses are presented in Supplementary Table S3 and Supplementary Table S4, respectively.

**Table 2:** PRO scores of all patients at all timepoints

Questionnaire	Baseline		After neoadjuvant treatment		3 months postoperatively		6 months postoperatively	
PRO, mean (SD)	Experimental arm	Control arm	Experimental arm	Control arm	Experimental arm	Control arm	Experimental arm	Control arm
*EQ-5D-5L*								
Index value	0.83 (0.17)	0.84 (0.18)	0.79 (0.22)	NA	0.79 (0.22)	0.77 (0.16)	0.86 (0.20)	0.83 (0.13)
Visual analog scale	77 (18)	75 (19)	66 (24)	NA	68 (22)	65 (28)	79 (14)	75 (12)
*EORTC QLQ-C30*								
Global health status	75 (16)	76 (20)	68 (16)	NA	70 (20)	70 (20)	77 (18)	72 (18)
Physical functioning	83 (18)	86 (16)	82 (18)	NA	77 (17)	74 (20)	86 (12)	79 (18)
Role functioning	76 (25)	77 (28)	67 (24)	NA	61 (27)	60 (28)	77 (24)	69 (25)
Emotional functioning	72 (20)	75 (19)	78 (22)	NA	84 (18)	76 (23)	85 (20)	77 (22)
Cognitive functioning	89 (17)	90 (12)	82 (22)	NA	88 (18)	82 (18)	85 (19)	82 (18)
Social functioning	86 (19)	80 (22)	75 (19)	NA	77 (23)	71 (25)	86 (17)	77 (27)
Fatigue	25 (19)	26 (19)	38 (27)	NA	41 (22)	38 (23)	25 (21)	30 (23)
Nausea/vomiting	2 (7)	5 (15)	6 (9)	NA	10 (20)	16 (30)	6 (12)	10 (17)
Pain	21 (22)	19 (24)	13 (21)	NA	21 (25)	27 (24)	11 (20)	20 (24)
Dyspnea	11 (19)	6 (15)	11 (18)	NA	17 (26)	23 (23)	10 (22)	16 (21)
Insomnia	19 (24)	24 (22)	24 (29)	NA	18 (28)	31 (28)	14 (23)	24 (25)
Loss of appetite	6 (17)	45 (37)	22 (26)	NA	23 (29)	36 (39)	21 (31)	26 (36)
Constipation	5 (14)	9 (22)	3 (10)	NA	9 (21)	9 (23)	0 (0)	8 (21)
Diarrhea	9 (22)	9 (18)	15 (23)	NA	15 (24)	17 (27)	10 (16)	18 (29)
Financial difficulties	8 (20)	6 (18)	11 (23)	NA	10 (23)	6 (15)	9 (19)	3 (10)
C30 summary score	85 (10)	82 (11)	81 (10)	NA	80 (13)	74 (15)	87 (11)	80 (13)
*EORTC QLQ-CR29*								
Urinary frequency	25 (23)	29 (21)	25 (24)	NA	25 (22)	31 (25)	23 (25)	29 (25)
Urinary incontinence	5 (12)	3 (16)	6 (19)	NA	4 (14)	12 (23)	6 (20)	16 (19)
Dysuria	5 (14)	3 (10)	1 (6)	NA	3 (8)	3 (9)	0 (0)	2 (9)
Abdominal pain	19 (18)	29 (27)	14 (19)	NA	20 (28)	24 (25)	14 (21)	22 (25)
Buttock pain	4 (10)	5 (14)	9 (19)	NA	4 (14)	10 (24)	2 (8)	16 (29)
Bloating ara>	12 (20)	15 (21)	11 (20)	NA	14 (22)	20 (27)	11 (20)	17 (26)
Blood/mucus in stool	1 (5)	5 (14)	2 (5)	NA	1 (3)	6 (10)	1 (3)	4 (10)
Dry mouth	9 (19)	9 (20)	23 (28)	NA	11 (18)	19 (25)	10 (16)	17 (23)
Hair loss	3 (12)	0 (0)	21 (28)	NA	12 (20)	13 (22)	10 (23)	9 (17)
Loss of taste	3 (12)	4 (13)	30 (35)	NA	19 (22)	19 (31)	11 (22)	14 (23)
Flatulence	15 (22)	20 (22)	24 (25)	NA	28 (24)	23 (23)	19 (24)	32 (26)
Fecal incontinence	3 (9)	7 (14)	6 (15)	NA	6 (18)	21 (27)	3 (10)	16 (27)
Sore skin	7 (16)	4 (11)	11 (20)	NA	5 (15)	15 (25)	7 (14)	16 (27)
Stool frequency	14 (21)	10 (20)	12 (16)	NA	9 (15)	21 (25)	99 (99)	99 (99)
Embarrassment	13 (29)	10 (19)	14 (26)	NA	13 (28)	28 (27)	11 (27)	24 (29)
Stoma care problems	25 (24)	4 (12)	21 (25)	NA	4 (12)	24 (34)	4 (12)	7 (18)
Impotence (m)	25 (33)	26 (38)	25 (33)	NA	21 (31)	42 (41)	29 (33)	44 (39)
Dyspareunia (f)	8 (21)	0 (0)	11 (16)	NA	13 (17)	11 (24)	14 (30)	0 (0)
Anxiety	44 (26)	46 (29)	57 (27)	NA	66 (26)	51 (28)	66 (22)	58 (30)
Weight	86 (24)	85 (21)	81 (25)	NA	83 (28)	80 (26)	82 (27)	88 (20)
Body image	83 (23)	89 (16)	82 (22)	NA	80 (21)	79 (22)	81 (21)	79 (19)
Sexual interest (m)	31 (26)	28 (21)	29 (21)	NA	31 (20)	19 (17)	40 (23)	26 (26)
Sexual interest (f)	15 (17)	12 (16)	15 (17)	NA	21 (17)	4 (12)	14 (17)	3 (10)

*NA* not applicable, *PRO* patient-reported outcome, *SD* standard deviation, *EORTC* European Organization for Research and Treatment of Cancer

### Primary Comparative Analyses

**Fig. 2:**
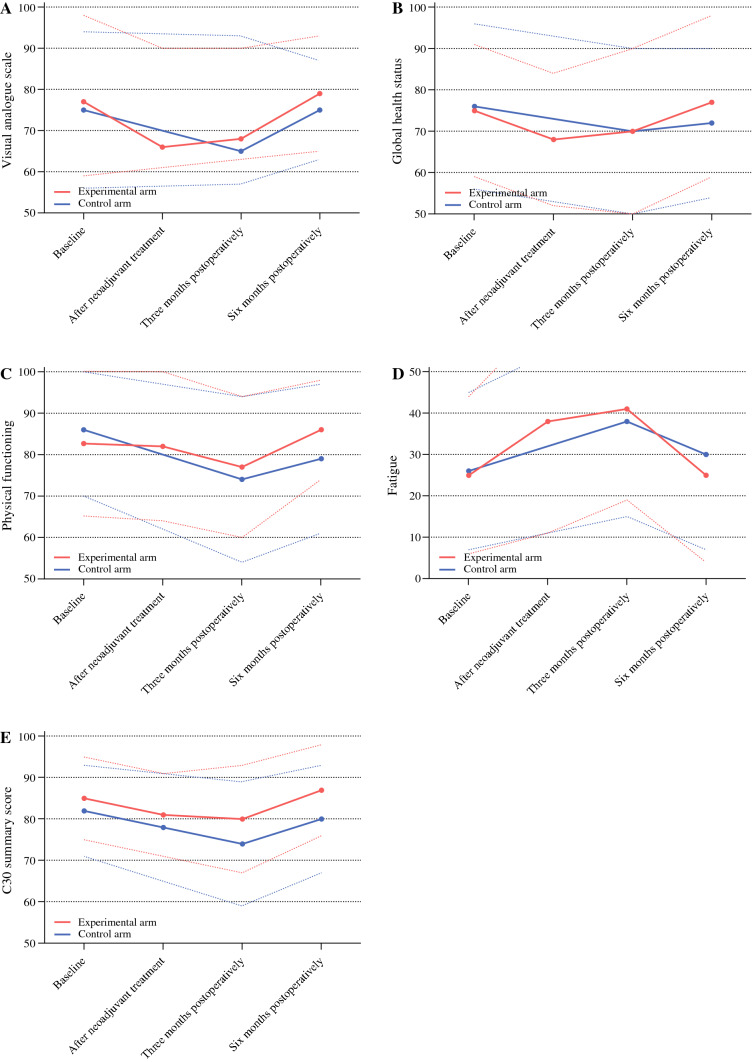
Primary comparison of five predefined PROs between both arms. Lines represent mean scores; dashed lines represent standard deviations

Figure 2 shows the primary comparisons of five predefined PROs between both arms, with corresponding LMM presented in Supplementary Table S5.

Visual analog scale. Differential effects over time (*p *= 0.315) and scores at each timepoint were comparable between both arms (Fig. 2A, Supplementary Table S5). Overall, compared with baseline, visual analog scale worsened at 3 months postoperatively [mean difference (MD) − 10, 95% confidence interval (CI) − 15 to − 4, *p *= 0.001, CD 0.42] and returned to baseline at 6 months postoperatively (*p *= 0.932) (Supplementary Table S5).

Global health status. Differential effects over time (*p *= 0.444) and scores at each time point were comparable between both arms (Fig. 2B, Supplementary Table S5). Overall, compared with baseline, global health status remained stable at 3 months postoperatively (*p *= 0.017) and at 6 months postoperatively (*p *= 0.479) (Supplementary Table S5).

Physical functioning. Differential effects over time (*p *= 0.460) and scores at each time point were comparable between both arms (Fig. 2C, Supplementary Table S5). Overall, compared with baseline, physical functioning worsened at 3 months postoperatively (MD − 9, 95% CI − 13 to − 6, *p* < 0.001, CD 0.50) and returned to baseline at 6 months postoperatively (*p *= 0.039) (Supplementary Table S5).

Fatigue. Differential effects over time (*p *= 0.642) and scores at each timepoint were comparable between both arms (Fig. 2D, Supplementary Table S5). Overall, compared with baseline, fatigue worsened at 3 months postoperatively (MD + 15, 95% CI 9 to 20, *p* < 0.001, CD 0.71) and returned to baseline at 6 months postoperatively (*p *= 0.345) (Supplementary Table S5).

C30 summary score. Differential effects over time (*p *= 0.033) and scores at each time point were comparable between both arms (Fig. 2E, Supplementary Table S5). Overall, compared with baseline, C30 summary score worsened at 3 months postoperatively (MD − 7, 95% CI − 10 to − 4, *p* < 0.001, CD 0.56) and returned to baseline at 6 months postoperatively (*p *= 0.482) (Supplementary Table S5).

### Secondary Explorative Analyses

**Fig. 3:**
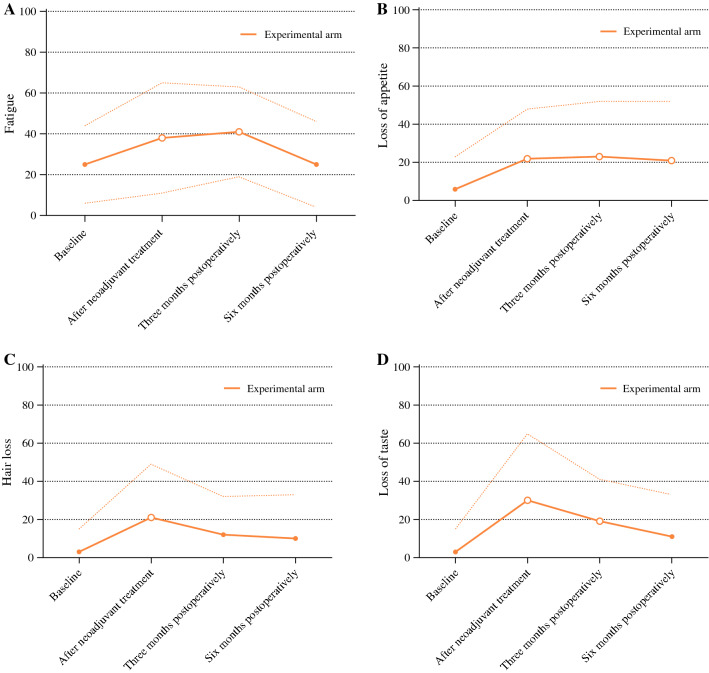
PROs with a statistically significant difference in scores between baseline and after neoadjuvant treatment in secondary explorative analyses in the experimental arm. Lines represent mean scores; dashed lines represent standard deviations; hollow dots indicate a statistically significant difference compared with baseline

Explorative LMM in the experimental arm showed that four PROs had a statistically significant difference in scores between baseline and after neoadjuvant treatment: fatigue, loss of appetite, hair loss, and loss of taste*.* Figure 3 shows these PROs, with corresponding LMM shown in Supplementary Table S6*.* All other PROs had no statistically significant difference in scores between baseline and after neoadjuvant treatment.

Fatigue. Fatigue differed over time (*p* < 0.001, Fig. 3A, Supplementary Table S6): compared with baseline, it worsened after neoadjuvant treatment (MD + 14, 95% CI 6–23, *p *= 0.001*,* CD 0.61), was still worse at 3 months postoperatively (MD + 17, 95% CI 9–26, *p* < 0.001, CD 0.85), and returned to baseline at 6 months postoperatively (*p *= 0.931).

Loss of appetite. Loss of appetite differed over time (*p* < 0.001, Fig. 3B, Supplementary Table S6): compared with baseline, it worsened after neoadjuvant treatment (MD + 15, 95% CI 5–25, *p *= 0.003, CD 0.67) and was still worse at 3 months postoperatively (MD + 16, 95% CI 6–29, *p *= 0.003, CD 0.66) and at 6 months postoperatively (MD + 14, 95% CI 4–25, *p* = 0.007*,* CD 0.55).

Hair loss. Hair loss differed over time (*p *= 0.002, Fig. 3C, Supplementary Table S6): compared with baseline, it worsened after neoadjuvant treatment (MD + 18, 95% CI 9–27, *p* < 0.001*,* CD 0.84), returned to baseline at 3 months postoperatively (*p *= 0.047) and at 6 months postoperatively (*p *= 0.105).

Loss of taste. Loss of taste differed over time (*p* < 0.001, Fig. 3D, Supplementary Table S6): compared with baseline, it worsened after neoadjuvant treatment (MD + 27, 95% CI 19–36, *p* < 0.001, CD 1.03), was still worse at 3 months postoperatively (MD + 16, 95% CI 7–25, *p *= 0.001, CD 0.90), and returned to baseline at 6 months postoperatively (*p *= 0.074).

## Discussion

In patients with resectable colorectal peritoneal metastases, randomized to perioperative systemic therapy or CRS–HIPEC alone, all predefined PROs (i.e., visual analog scale, global health status, physical functioning, fatigue, C30 summary score) were comparable between both arms at baseline and 3 and 6 months postoperatively. These PROs returned to baseline at 3 or 6 months postoperatively in both arms. Secondary explorative analyses in the experimental arm showed statistically significant and clinically relevant worsening of fatigue, hair loss, loss of taste, and loss of appetite after neoadjuvant treatment. Except for loss of appetite, these PROs returned to baseline at 3 or 6 months postoperatively.

To the knowledge of the authors, the present study is the first to compare PROs between perioperative systemic therapy or CRS–HIPEC alone for resectable colorectal peritoneal metastases. Findings of the present study provide relevant insight in the burden of perioperative systemic therapy in this setting and show acceptable treatment tolerability. Together with the previously demonstrated safety and feasibility of perioperative systemic therapy in patients with resectable colorectal peritoneal metastases,^8^ results of the present study justify a phase III trial and may facilitate its informed consent. To the knowledge of the authors, PROs have also never been compared between perioperative systemic therapy and surgery alone in patients with other malignancies. As a result, findings of the present study may also be valuable for physicians administering similar perioperative systemic regimens to patients with other malignancies that require extensive surgery.

A recent systematic review identified 14 other studies reporting PROs in patients undergoing CRS–HIPEC.^17^ However, none of these studies specifically focused on perioperative systemic therapy and its possible effect on PROs.^17^ Nevertheless, the only two studies specifically focusing on PROs after CRS–HIPEC for colorectal peritoneal metastases reported postoperative recovery times of PROs similar to the present study.^18,19^

The present study showed worsening of fatigue, loss of appetite, hair loss, and loss of taste after neoadjuvant treatment. Although these symptoms are generally recognized as common observer-reported side effects of systemic therapy for colorectal cancer in clinical trials,^20^ PROs after neoadjuvant treatment for (potentially) resectable metastatic colorectal cancer have never been reported. While all the worsening PROs after neoadjuvant treatment (except for loss of appetite) returned to baseline levels at 3 or 6 months postoperatively, patients in the experimental arm underwent treatment for a considerably longer period than patients in the control arm. Thereby, they may have experienced a longer period of worsened PROs. Nevertheless, the present study suggests that these worsening PROs after neoadjuvant treatment did not translate into a postoperative difference in five predefined general PROs between both arms, even though many patients in the experimental arm received adjuvant treatment at time of the first postoperative PRO measurement at three months postoperatively. Several factors may explain the absence of differences in these predefined postoperative PROs between both arms. First, patients’ psychological adaptation to their changing health status over time, a phenomenon called response shift, could have contributed to the lack of worsening of postoperative PROs in the experimental arm despite toxicity of perioperative systemic therapy.^21^ Second, patients receiving perioperative systemic therapy may have an increased belief in cure,^22^ as this is the hypothesis of the CAIRO6 trial. Third, patients could have had the perception that side effects of perioperative systemic therapy are a sign of treatment efficacy.^22^ The latter two factors may have counteracted the possible negative effects of perioperative systemic therapy and its toxicity on PROs in the experimental arm.

The main strength of the present study is the overall response rate of 87%, which is high compared with other PRO studies: 65% in a randomized trial of neoadjuvant chemoradiotherapy and surgery versus surgery alone in esophageal cancer, and 65% in a systematic review of metastatic colorectal cancer trials.^21,23^ Given the severity of the disease and the treatment intensity, the authors expected a higher chance of bias due to drop-outs. Nevertheless, unavoidable drop-out of the most severely ill patients during the trial could have overestimated PRO scores at 3 and 6 months postoperatively in both groups. As the drop-out percentages did not differ between both groups (chi-square* p *= 0.307, data not shown), the authors conclude that a comparison between both groups can still be made and well interpreted. The main limitation of the present study is the relatively small sample size of 80 patients. Though LMM allowed detection of both statistically significant and clinically relevant differences, a larger sample size could have detected additional statistically significant fluctuations in PROs that may have been clinically relevant.

## Conclusions

In patients with resectable colorectal peritoneal metastases randomized to perioperative systemic therapy or CRS–HIPEC alone, all predefined PROs were comparable between both arms and returned to baseline at 3 or 6 months postoperatively. Though several PROs worsened after neoadjuvant treatment, all of these (except for loss of appetite) returned to baseline at 3 or 6 months postoperatively. Together with the trial’s previously reported feasibility and safety data, these findings show acceptable tolerability of perioperative systemic therapy in this setting and justify a phase III trial.

## Supplementary Information

Below is the link to the electronic supplementary material.

Supplementary file1 (DOCX 56 kb)
