# Supplementary material for: Perioperative Systemic Therapy Versus Cytoreductive Surgery and HIPEC Alone for Resectable Colorectal Peritoneal Metastases: Patient-Reported Outcomes of a Randomized Phase II Trial
Source: Ann Surg Oncol. 2023 Feb 8;30(5):2678–88. doi: 10.1245/s10434-023-13116-z (PMC10085918; doi:10.1245/s10434-023-13116-z)
Supplement: Supplementary file 1 — Supplementary file1 (DOCX 56 kb) [file 10434_2023_13116_MOESM1_ESM.docx]

**Supplementary Table S1.** PROs of each questionnaire.

|  |  |  |
| --- | --- | --- |
| **Questionnaire** | **PROs – Function scales^a^** | **PROs – Symptom scales^b^** |
| EQ-5D-5L | Visual analogue scale; Index value | - |
| EORTC QLQ-C30 | Global health status; Physical functioning; Role functioning; Emotional functioning; Cognitive functioning; Social functioning, C30 summary score | Fatigue; Nausea and vomiting; Pain; Dyspnea; Insomnia; Loss of appetite; Constipation; Diarrhea; Financial difficulties |
| EORTC QLQ-CR29 | Weight; Body image; Sexual interest (males); Sexual interest (females) | Urinary frequency; Urinary incontinence; Dysuria; Abdominal pain; Buttock pain; Bloating; Blood and mucus in stool; Dry mouth; Hair loss; Loss of taste; Flatulence; Fecal incontinence; Sore skin; Stool frequency; Embarrassment; Stoma care problems; Impotence (males); Dyspareunia (females); Anxiety |
| *PROs* patient-reported outcomes; *EORTC* European organization for research and treatment of cancer; ^a^higher score indicates better functioning; ^b^higher score indicates worse symptoms. | | |

**Supplementary Table S2.** Baseline characteristics of the intention-to-treat trial population.

|  |  |  |  |
| --- | --- | --- | --- |
|  | **Experimental (n=40)** | **Control**  **(n=40)** | **P-value** |
| **Sex, *n* (%)** |  |  | 0.262 |
| Male | 19 (48) | 24 (60) |  |
| Female | 21 (52) | 16 (40) |  |
| **Age in years, mean (SD)** | 60 (11) | 64 (10) | **0.047** |
| **WHO performance score**, ***n* (%)** |  |  | 0.587 |
| 0 | 30 (75) | 33 (83) |  |
| 1 | 9 (23) | 7 (17) |  |
| 2 | 1 (3)^a^ | 0 (0) |  |
| **Primary tumor location, *n* (%)** |  |  | 0.742 |
| Proximal colon^b^ | 17 (43) | 14 (35) |  |
| Distal colon^c^ | 21 (53) | 25 (63) |  |
| Rectum | 1 (3) | 1 (3) |  |
| Multiple | 1 (3) | 0 (0) |  |
| **Primary tumor status, *n* (%)** |  |  | 0.160 |
| Resected | 30 (75) | 23 (57) |  |
| In situ | 10 (25) | 17 (43) |  |
| **Previous adjuvant systemic chemotherapy for colorectal cancer, *n* (%)** |  |  | 0.606 |
| No | 29 (73) | 31 (78) |  |
| Yes | 11 (27) | 9 (23) |  |
| **Onset of peritoneal metastases, *n* (%)** |  |  | 0.499 |
| Synchronous | 21 (53) | 24 (60) |  |
| Metachronous | 19 (48) | 16 (40) |  |
| **Baseline peritoneal cancer index, median (range)** | 3 (0-15) | 5 (1-18) | 0.064 |
| **Planned HIPEC regimen, *n* (%)** |  |  | >0.999 |
| Mitomycin C | 32 (80) | 32 (80) |  |
| Oxaliplatin | 8 (20) | 8 (20) |  |
| *HIPEC* hyperthermic intraperitoneal chemotherapy; *SD* standard deviation; *WHO* world health organization; ^a^due to severe obesity; ^b^caecum, ascending colon, hepatic flexure, transverse colon; ^c^splenic flexure, descending colon, sigmoid, rectosigmoid. | | | |

**Supplementary Table S3.** PRO scores of 68 patients included in primary comparative analyses.

| **Questionnaire** | **Baseline** | | **After neoadjuvant treatment** | | **Three months postoperatively** | | **Six months**  **postoperatively** | |
| --- | --- | --- | --- | --- | --- | --- | --- | --- |
| **PRO, mean (SD)** | **Experimental**  **arm** | **Control**  **arm** | **Experimental**  **arm** | **Control arm** | **Experimental arm** | **Control arm** | **Experimental arm** | **Control arm** |
| **EQ-5D-5L** | | | | | | | | |
| Index value | 0.85 (0.16) | 0.84 (0.19) | 0.79 (0.22) | NA | 0.79 (0.22) | 0.77 (0.16) | 0.86 (0.20) | 0.82 (0.13) |
| Visual analogue scale | 77 (19) | 75 (20) | 68 (22) | NA | 68 (22) | 65 (28) | 79 (14) | 74 (12) |
| **EORTC QLQ-C30** | | | | | | | | |
| Global health status | 77 (16) | 75 (20) | 69 (16) | NA | 70 (20) | 70 (20) | 77 (18) | 72 (18) |
| Physical functioning | 85 (17) | 85 (17) | 82 (16) | NA | 77 (17) | 74 (20) | 86 (12) | 79 (19) |
| Role functioning | 74 (26) | 76 (29) | 66 (25) | NA | 61 (27) | 60 (28) | 77 (24) | 69 (26) |
| Emotional functioning | 75 (18) | 75 (18) | 78 (22) | NA | 84 (18) | 76 (23) | 85 (20) | 76 (22) |
| Cognitive functioning | 89 (17) | 89 (12) | 83 (22) | NA | 88 (18) | 82 (18) | 85 (19) | 82 (18) |
| Social functioning | 84 (19) | 76 (20) | 81 (22) | NA | 77 (23) | 71 (25) | 86 (17) | 76 (27) |
| Fatigue | 24 (19) | 26 (19) | 39 (27) | NA | 41 (22) | 38 (23) | 25 (21) | 30 (23) |
| Nausea/vomiting | 2 (7) | 6 (16) | 5 (9) | NA | 10 (20) | 16 (30) | 6 (12) | 10 (17) |
| Pain | 23 (23) | 18 (24) | 13 (21) | NA | 21 (25) | 27 (24) | 11 (20) | 21 (24) |
| Dyspnea | 8 (17) | 06 (16) | 11 (18) | NA | 17 (26) | 23 (23) | 10 (22) | 16 (21) |
| Insomnia | 19 (24) | 27 (22) | 25 (30) | NA | 18 (28) | 31 (28) | 14 (23) | 25 (25) |
| Loss of appetite | 6 (18) | 41 (36) | 22 (27) | NA | 23 (29) | 36 (39) | 21 (31) | 24 (36) |
| Constipation | 5 (15) | 7 (18) | 1 (6) | NA | 9 (21) | 9 (23) | 0 (0) | 8 (21) |
| Diarrhea | 8 (22) | 10 (19) | 16 (24) | NA | 15 (24) | 17 (27) | 10 (16) | 16 (28) |
| Financial difficulties | 9 (21) | 6 (19) | 12 (24) | NA | 10 (23) | 6 (15) | 9 (20) | 3 (10) |
| C30 summary score | 86 (11) | 82 (11) | 81 (10) | NA | 80 (13) | 74 (15) | 87 (11) | 80 (13) |
| **EORTC QLQ-CR29** | | | | | | | | |
| Urinary frequency | 25 (22) | 30 (21) | 25 (24) | NA | 25 (22) | 31 (25) | 23 (25) | 29 (25) |
| Urinary incontinence | 4 (11) | 9 (17) | 06 (20) | NA | 4 (14) | 12 (23) | 6 (20) | 15 (19) |
| Dysuria | 4 (14) | 4 (11) | 1 (6) | NA | 2 (8) | 3 (9) | 0 (0) | 2 (6) |
| Abdominal pain | 19 (19) | 29 (28) | 14 (19) | NA | 20 (28) | 24 (25) | 14 (21) | 23 (25) |
| Buttock pain | 3 (10) | 6 (15) | 9 (19) | NA | 4 (14) | 10 (24) | 2 (8) | 14 (27) |
| Bloating | 10 (20) | 15 (20) | 10 (18) | NA | 14 (22) | 20 (27) | 11 (20) | 16 (26) |
| Blood/mucus in stool | 2 (5) | 6 (15) | 2 (5) | NA | 5 (3) | 6 (10) | 1 (3) | 4 (10) |
| Dry mouth | 8 (17) | 8 (18) | 22 (28) | NA | 11 (18) | 19 (25) | 10 (16) | 17 (23) |
| Hair loss | 3 (13) | 0 (0) | 19 (28) | NA | 12 (20) | 13 (22) | 10 (23) | 9 (18) |
| Loss of taste | 3 (13) | 04 (13) | 32 (36) | NA | 19 (22) | 19 (31) | 11 (22) | 15 (23) |
| Flatulence | 16 (22) | 21 (23) | 24 (26) | NA | 28 (24) | 23 (23) | 19 (24) | 31 (26) |
| Fecal incontinence | 1 (6) | 6 (13) | 6 (16) | NA | 6 (18) | 21 (27) | 3 (10) | 16 (28) |
| Sore skin | 7 (16) | 4 (11) | 11 (20) | NA | 5 (15) | 15 (25) | 7 (14) | 15 (28) |
| Stool frequency | 15 (21) | 11 (20) | 13 (17) | NA | 9 (15) | 21 (25) | 11 (17) | 22 (26) |
| Embarrassment | 14 (30) | 9 (19) | 14 (27) | NA | 13 (28) | 28 (27) | 11 (27) | 25 (29) |
| Stoma care problems | 24 (25) | 7 (15) | 19 (26) | NA | 4 (12) | 24 (34) | 4 (12) | 8 (19) |
| Impotence (m) | 29 (34) | 25 (37) | 29 (34) | NA | 21 (31) | 42 (41) | 29 (33) | 43 (39) |
| Dyspareunia (f) | 8 (21) | 0 (0) | 12 (17) | NA | 13 (17) | 11 (24) | 14 (30) | 0 (0) |
| Anxiety | 46 (25) | 44 (30) | 59 (25) | NA | 65 (26) | 51 (28) | 66 (22) | 56 (30) |
| Weight | 88 (25) | 85 (22) | 81 (25) | NA | 83 (28) | 80 (26) | 82 (27) | 87 (21) |
| Body image | 85 (22) | 91 (13) | 81 (22) | NA | 80 (21) | 79 (22) | 81 (21) | 79 (19) |
| Sexual interest (m) | 36 (24) | 28 (22) | 31 (21) | NA | 31 (20) | 19 (17) | 40 (23) | 26 (27) |
| Sexual interest (f) | 17 (17) | 10 (16) | 16 (17) | NA | 21 (17) | 04 (12) | 14 (17) | 3 (10) |
| *NA* not applicable; *PRO* patient-reported outcome; *SD* standard deviation; *EORTC* European organization for research and treatment of cancer. | | | | | | | | |

**Supplementary Table S4.** PRO scores of 35 patients of the experimental arm included in secondary longitudinal explorative analyses.

| **PRO, mean (SD)** | **Baseline** | **After neoadjuvant treatment** | **Three months postoperatively** | **Six months postoperatively** |
| --- | --- | --- | --- | --- |
| **EQ-5D-5L** | | | | |
| Index value | 0.84 (0.16) | 0.79 (0.22) | 0.79 (0.22) | 0.86 (0.20) |
| Visual analogue scale | 77 (18) | 66 (24) | 68 (22) | 79 (14) |
| **EORTC QLQ-C30** | | | | |
| Global health status | 76 (15) | 68 (16) | 70 (20) | 77 (18) |
| Physical functioning | 84 (18) | 80 (18) | 77 (17) | 86 (12) |
| Role functioning | 75 (25) | 67 (24) | 61 (27) | 77 (24) |
| Emotional functioning | 74 (18) | 78 (22) | 84 (18) | 85 (20) |
| Cognitive functioning | 89 (17) | 82 (22) | 88 (18) | 85 (19) |
| Social functioning | 85 (19) | 75 (19) | 77 (23) | 86 (17) |
| Fatigue | 24 (18) | 38 (27) | 41 (22) | 25 (21) |
| Nausea/vomiting | 2 (7) | 6 (9) | 10 (20) | 6 (12) |
| Pain | 22 (22) | 13 (21) | 21 (25) | 11 (20) |
| Dyspnea | 9 (17) | 11 (18) | 17 (26) | 10 (22) |
| Insomnia | 20 (25) | 24 (29) | 18 (28) | 14 (23) |
| Loss of appetite | 7 (18) | 22 (26) | 23 (29) | 21 (31) |
| Constipation | 5 (14) | 3 (10) | 9 (21) | 0 (0) |
| Diarrhea | 10 (22) | 15 (23) | 15 (24) | 10 (16) |
| Financial difficulties | 9 (20) | 11 (23) | 10 (23) | 9 (19) |
| C30 summary score | 85 (11) | 81 (10) | 80 (13) | 87 (11) |
| **EORTC QLQ-CR29** | | | | |
| Urinary frequency | 26 (23) | 25 (24) | 25 (22) | 23 (25) |
| Urinary incontinence | 5 (12) | 6 (19) | 4 (14) | 6 (20) |
| Dysuria | 5 (14) | 1 (6) | 2 (8) | 0 (0) |
| Abdominal pain | 19 (19) | 14 (19) | 20 (28) | 14 (21) |
| Buttock pain | 4 (11) | 9 (19) | 4 (14) | 2 (8) |
| Bloating | 12 (20) | 11 (20) | 14 (22) | 11 (20) |
| Blood/mucus in stool | 1 (5) | 2 (5) | 1 (3) | 1 (3) |
| Dry mouth | 10 (19) | 23 (28) | 11 (18) | 10 (16) |
| Hair loss | 3 (12) | 21 (28) | 12 (20) | 10 (23) |
| Loss of taste | 3 (12) | 30 (35) | 19 (22) | 11 (22) |
| Flatulence | 15 (22) | 24 (25) | 28 (24) | 19 (24) |
| Fecal incontinence | 2 (8) | 6 (15) | 6 (18) | 3 (10) |
| Sore skin | 8 (16) | 11 (20) | 5 (15) | 7 (14) |
| Stool frequency | 14 (21) | 12 (16) | 9 (15) | 11 (17) |
| Embarrassment | 13 (29) | 14 (26) | 13 (28) | 11 (27) |
| Stoma care problems | 25 (24) | 21 (25) | 4 (12) | 4 (12) |
| Impotence (m) | 25 (33) | 25 (33) | 21 (31) | 29 (33) |
| Dyspareunia (f) | 8 (21) | 11 (16) | 13 (17) | 14 (30) |
| Anxiety | 45 (25) | 57 (27) | 65 (26) | 66 (22) |
| Weight | 87 (25) | 81 (25) | 83 (28) | 82 (27) |
| Body image | 85 (21) | 82 (22) | 80 (21) | 81 (21) |
| Sexual interest (m) | 31 (26) | 29 (21) | 31 (20) | 40 (23) |
| Sexual interest (f) | 16 (17) | 15 (17) | 21 (17) | 14 (17) |
| *PRO* patient-reported outcome; *SD* standard deviation; *EORTC* European organization for research and treatment of cancer. | | | | |

**Supplementary Table S5.** Primary comparative analyses of five predefined PROs between both arms using linear mixed modeling.

|  |  |  |  |  |
| --- | --- | --- | --- | --- |
| **PRO** | **Mean difference** | **95% CI** | **P-value** | **Cohen’s d^a^** |
| **Visual analogue scale** | | | | |
| Between-arm comparison of differential effect in scores over time | - | - | 0.315 | - |
| Between-arm comparison of scores at each time point |  |  |  |  |
| Baseline | +2^b^ | -8 to 11 | 0.687 | - |
| Three months postoperatively | +3^b^ | -7 to 12 | 0.574 | - |
| Six months postoperatively | +5^b^ | -3 to 17 | 0.191 | - |
| Longitudinal analyses after merging scores of both arms |  |  |  |  |
| Baseline vs. three months postoperatively | -10^c^ | -15 to -4 | 0.001 | 0.42 |
| Baseline vs. six months postoperatively | +1^c^ | -5 to 6 | 0.932 | - |
| **Global health status** | | | | |
| Between-arm comparison of differential effect in scores over time | - | - | 0.444 | - |
| Between-arm comparison of scores at each time point |  |  |  |  |
| Baseline | +1^b^ | -7 to 11 | 0.656 | - |
| Three months postoperatively | +0^b^ | -9 to 9 | 0.949 | - |
| Six months postoperatively | +5^b^ | -3 to 16 | 0.176 | - |
| Longitudinal analyses after merging scores of both arms |  |  |  |  |
| Baseline vs. three months postoperatively | -6^c^ | -11 to -1 | 0.017 | - |
| Baseline vs. six months postoperatively | -1^c^ | -7 to 3 | 0.479 | - |
| **Physical functioning** | | | | |
| Between-arm comparison of differential effect in scores over time | - | - | 0.460 | - |
| Between-arm comparison of scores at each time point |  |  |  |  |
| Baseline | +0^b^ | -9 to 8 | 0.886 | - |
| Three months postoperatively | +3^b^ | -6 to 11 | 0.520 | - |
| Six months postoperatively | +7^b^ | -2 to 16 | 0.122 | - |
| Longitudinal analyses after merging scores of both arms |  |  |  |  |
| Baseline vs. three months postoperatively | -9^c^ | -13 to -6 | <0.001 | 0.50 |
| Baseline vs. six months postoperatively | -2^c^ | -8 to 0 | 0.039 | - |
| **Fatigue** | | | | |
| Between-arm comparison of differential effect in scores over time | - | - | 0.642 | - |
| Between-arm comparison of scores at each time point |  |  |  |  |
| Baseline | -2^b^ | -13 to 7 | 0.608 | - |
| Three months postoperatively | +3^b^ | -7 to 13 | 0.553 | - |
| Six months postoperatively | -5^b^ | -17 to 4 | 0.213 | - |
| Longitudinal analyses after merging scores of both arms |  |  |  |  |
| Baseline vs. three months postoperatively | +15^c^ | 9 to 20 | <0.001 | 0.71 |
| Baseline vs. six months postoperatively | +3^c^ | -3 to 8 | 0.345 | - |
| **C30 summary score** | | | | |
| Between-arm comparison of differential effect in scores over time | - | - | 0.033 | - |
| Between-arm comparison of scores at each time point |  |  |  |  |
| Baseline | +4^b^ | -3 to 9 | 0.260 | - |
| Three months postoperatively | +6^b^ | -1 to 11 | 0.103 | - |
| Six months postoperatively | +7^b^ | 1 to 13 | 0.022 | - |
| Longitudinal analyses after merging scores of both arms |  |  |  |  |
| Baseline vs. three months postoperatively | -7^c^ | -10 to -4 | <0.001 | 0.56 |
| Baseline vs. six months postoperatively | +0^c^ | -4 to 2 | 0.482 | - |
| *CI* confidence interval; *PRO* patient-reported outcome; ^a^mean difference divided by pooled standard deviation; ^b^score of experimental arm minus score of control arm; ^c^score of last mentioned time point minus baseline score. | | | | |

**Supplementary Table S6.** Linear mixed modeling analyses of PROs with a statistically significant difference in score between baseline and after neoadjuvant treatment in the experimental arm.

|  | | | | |
| --- | --- | --- | --- | --- |
| **PRO** | **Mean difference^a^** | **95% CI** | **p-value** | **Cohen’s d^b^** |
| **Fatigue** | | | | |
| Differential effect in scores over time | - | - | <0.001 | - |
| Baseline vs. after neoadjuvant treatment | +14 | 6 to 23 | 0.001 | 0.61 |
| Baseline vs. three months postoperatively | +17 | 9 to 26 | <0.001 | 0.85 |
| Baseline vs. six months postoperatively | +1 | -8 to 9 | 0.931 | - |
| **Loss of appetite** | | | | |
| Differential effect in scores over time | - | - | 0.005 | - |
| Baseline vs. after neoadjuvant treatment | +15 | 5 to 25 | 0.003 | 0.67 |
| Baseline vs. three months postoperatively | +16 | 6 to 29 | 0.003 | 0.66 |
| Baseline vs. six months postoperatively | +14 | 4 to 25 | 0.007 | 0.55 |
| **Hair loss** | | | | |
| Differential effect in scores over time | - | - | 0.002 | - |
| Baseline vs. after neoadjuvant treatment | +18 | 9 to 26 | <0.001 | 0.84 |
| Baseline vs. three months postoperatively | +9 | 0 to 18 | 0.047 | - |
| Baseline vs. six months postoperatively | +7 | -1 to 17 | 0.105 | - |
| **Loss of taste** | | | | |
| Differential effect in scores over time | - | - | <0.001 | - |
| Baseline vs. after neoadjuvant treatment | +27 | 19 to 36 | <0.001 | 1.03 |
| Baseline vs. three months postoperatively | +16 | 7 to 25 | 0.001 | 0.90 |
| Baseline vs. six months postoperatively | +8 | -1 to 18 | 0.074 | - |
| **C30 summary score** | | | | |
| Differential effect in scores over time | - | - | 0.001 | - |
| Baseline vs. after neoadjuvant treatment | -4 | -8 to 0 | 0.021 | - |
| Baseline vs. three months postoperatively | -5 | -10 to -2 | 0.001 | 0.42 |
| Baseline vs. six months postoperatively | +2 | -3 to 5 | 0.672 | - |
| *CI* confidence interval; *PRO* patient-reported outcome; ^a^score of last mentioned time point minus baseline score; ^b^mean difference divided by pooled standard deviation. | | | | |
